# Supplementary material for: qPCR Detection and Quantification of Aggregatibacter actinomycetemcomitans and Other Periodontal Pathogens in Saliva and Gingival Crevicular Fluid among Periodontitis Patients
Source: Pathogens. 2023 Jan 3;12(1):76. doi: 10.3390/pathogens12010076 (PMC9861831; doi:10.3390/pathogens12010076)
Supplement: Supplementary file 1 [file pathogens-12-00076-s001.zip › pathogens-2054682-supplementary.pdf]

qPCR detection and quantification of *Aggregatibacter actinomycetemcomitans* and other periodontal pathogens in saliva and gingival crevicular fluid among periodontitis patients - Supplementary Data Sheet

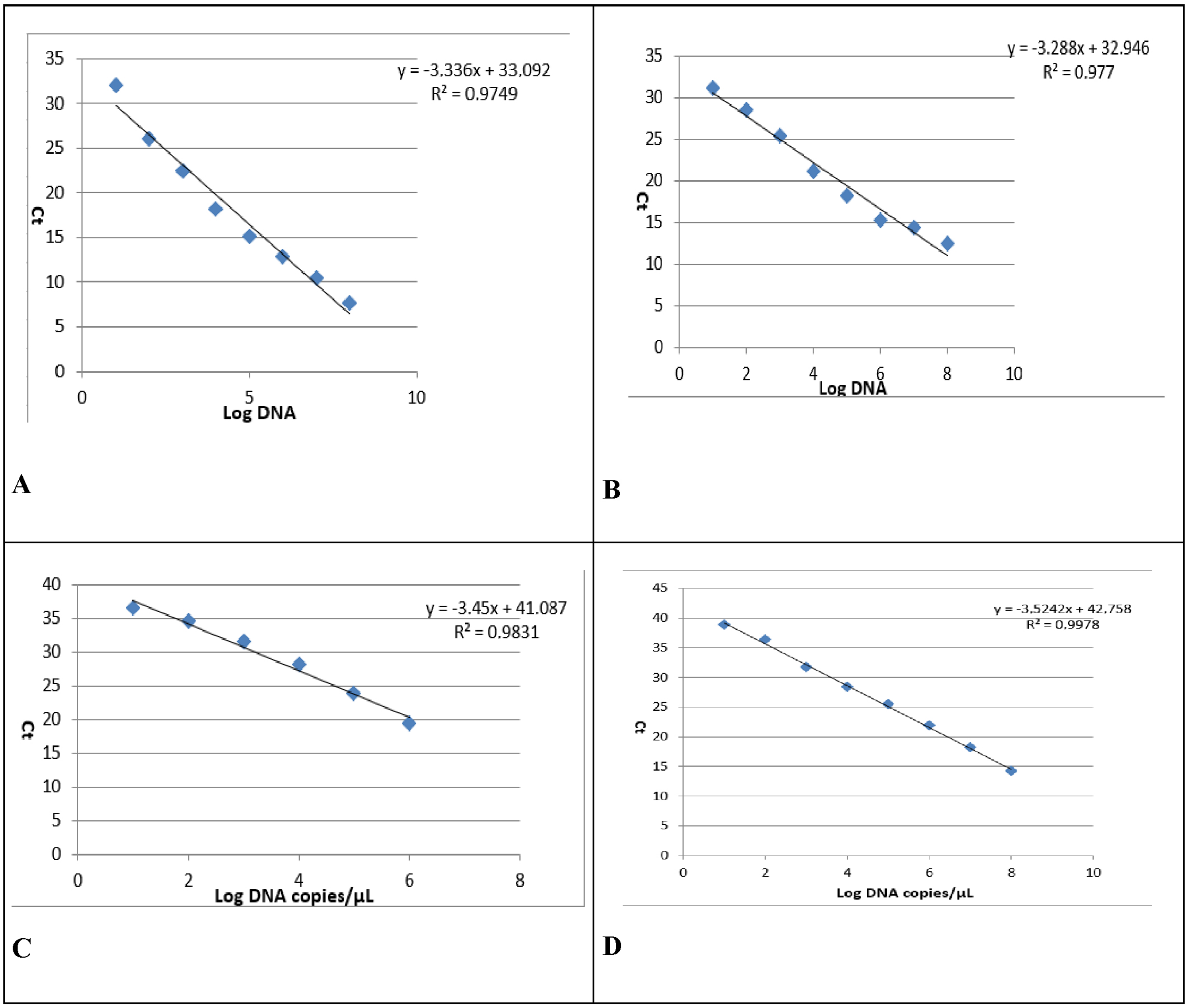

Figure 1: Standard curves of genomic DNA from *A. actinomycetemcomitans non JP2* (A), *A. actinomycetemcomitans JP2* (B), *P. gingivalis* (C) and Universal (D). Ct is the cycle number at which the threshold fluorescence is reached. The standard curves were generated from the amplification plots in the insets (correlation coefficients: 0.974 for *A. actinomycetemcomitans JP2* clone, 0.977 for *A. actinomycetemcomitans non JP2* clone, 0.983 for *P. gingivalis*, and 0.997 for Universal).
